# The COVID-19 pandemic reduced the trauma incidence and modified its pattern in Al-Ain City, United Arab Emirates

**DOI:** 10.1007/s00068-022-01897-z

**Published:** 2022-02-18

**Authors:** David Olukolade Alao, Arif Alper Cevik, Yasin Jemal Yasin, Thiagarajan Jaiganesh, Fikri Abu-Zidan

**Affiliations:** 1 Department of Internal Medicine, College of Medicine and Health Sciences, UAE University, Al-Ain, United Arab Emirates; 2 Emergency Department, Tawam Hospital, Al-Ain, United Arab Emirates; 3 Institute of Public Health, College of Medicine and Health Sciences, UAE University, Al-Ain, United Arab Emirates; 4 Department of Surgery, College of Medicine and Health Sciences, UAE University, Al-Ain, United Arab Emirates

**Keywords:** COVID-19, Trauma, Injury, Epidemiology, Death, United Arab Emirates

## Abstract

**Aim:**

We aimed to study the impact of the COVID-19 pandemic on the pattern of injury and outcome of hospitalized trauma patients in Al-Ain City, United Arab Emirates, to use this information in the preparedness for future pandemics.

**Methods:**

We retrospectively compared the trauma registry data of all hospitalised trauma patients, who were treated at the two main trauma centres in Al-Ain City (Al-Ain Hospital and Tawam Hospital); those who were treated over 1 year before the pandemic (*n* = 2002) and those who were treated at the first year of the COVID-19 pandemic (*n* = 1468).

**Results:**

There was a 26.7% reduction in the overall incidence of trauma admissions in the COVID-19 pandemic period. The mechanism of injury significantly differed between the two periods (*p* < 0.0001, Fisher’s exact test). There was an absolute increase in the number of injuries, due to machinery and falling objects during the pandemic (39.7% and 54.1% respectively, *p* < 0.001). In contrast, road traffic collisions and falls were reduced by 33.5% and 31.3%, respectively. Location significantly differed between the two periods (*p* < 0.0001, Fisher’s exact test). There was an absolute increase of 18.4% in workplace injuries and a reduction of 39.3% in home injuries over the study period. In addition, we observed relatively more workplace injuries and fewer home injuries during the pandemic (11.3% and 42.8% compared with 7.1% and 52.4%, respectively). Mortality was similar between the two periods (1.8% compared with 1.2%, *p* = 0.16, Fisher’s exact test).

**Conclusions:**

The COVID-19 pandemic has modified the trauma risk exposure in our population. It reduced trauma hospital admissions by around 27%. Work-related injuries, including falling objects and machinery injuries, were relatively higher during the pandemic. Prevention of work-related injuries should be an important component of preparedness for future pandemics.

## Introduction

Major trauma accounts for 5.8 million deaths annually and is the leading cause of death among children and young adults below 44 years [1]. Trauma is the second leading cause of death in the United Arab Emirates (UAE) behind cardio-vascular diseases. In 2017, it accounted for 17.2% of all deaths in the UAE, with motor vehicle collisions and falls from a height constituting over 70% of these deaths [2].

The COVID-19 pandemic started in 2019 in Wuhan, China. On 11th March 2020, the WHO declared it a worldwide pandemic. This has a significant impact on healthcare provision and resource utilisation, including trauma care. The first case of COVID-19 was confirmed in the UAE on 29th January 2020. By March 2020, several thousands of people had been infected by the virus. This necessitated a declaration of total lockdown by the UAE government.

Studies across the globe have reported a significant reduction in the incidence of trauma during the COVID-19 pandemic [3–6]. The lockdown imposed by different governments has reduced motor vehicle collision injuries, while interpersonal violence has increased due to more direct contact at home. We have shown recently that the COVID-19 lockdown reduced the road traffic collision (RTC) incidence worldwide, while increasing its severity. This was attributed to empty lanes, increased speed, and drivers ignoring traffic regulations [7, 8]. We anticipated that the COVID-19 pandemic impact on trauma outcomes might be different in our population. We aimed to study the impact of the COVID-19 pandemic on the pattern of injury and outcome of hospitalized trauma patients in Al-Ain City, UAE, to inform the preparedness for future pandemics.

## Patients and methods

### Ethical consideration

The Department of Health, Abu Dhabi Institutional Review Body, gave ethical approval for this study (Ref: DOH/CVDC/2021/650). The Al-Ain Hospital and Tawam Hospital institutional review body approved the study.

### Setting

Al-Ain Region of Abu Dhabi, United Arab Emirates, has an estimated population of 766,000. It had two major hospitals that received trauma emergencies prior to the pandemic. On 28th March 2020, following the outbreak of COVID-19, the hospitals were redesignated as COVID-19 and non-COVID-19. Al-Ain Hospital, which received the majority of the trauma patients before the outbreak, became the COVID-19 hospital. In contrast, Tawam Hospital was designated a non-COVID hospital and the only trauma receiving hospital. All trauma patients either self-presented or were brought by the emergency medical service to Tawam Hospital. The patients were managed and stabilised in the emergency department until the result of their SARS-COV-19 reverse transcriptase-polymerase chain reaction (RT PCR) test was known. Patients with a positive result were transferred to the COVID-19 hospital for ongoing care. If a patient needed an emergency life-saving operation, they would be operated on without the PCR test and kept in the recovery room of the operating theatre until the PCR result was received. This would take around 4 h. Patients would be admitted to Tawam Hospital if the result was negative; otherwise, they would be transferred to Al-Ain Hospital for further management.

### Patients

We studied all trauma patients who died in the hospital or who were admitted for more than 24 h at both Al-Ain and Tawam Hospital from 28th March 2019 to 27th March 2020 (pre-pandemic period) and those who were admitted to Tawam Hospital from 28th March 2020 to 27th March 2021 (pandemic period).

### Data collection

We extracted, from the Abu Dhabi trauma registry, anonymised data of all trauma patients who were admitted and treated at Tawam and Al-Ain Hospital over the study period. The Abu Dhabi trauma registry started in 2013 and was based on the national trauma data bank of the American College of Surgeons. It prospectively collects data on all trauma patients who died in the hospital or had been admitted for more than 24 h.

### Studied variables

We compared the two periods regarding patients’ demographics, mechanisms of injury, locations of injury, physiological and anatomical severity indicators, ISS, hospital and ICU length of stay, and clinical outcome, including death.

### Statistical analysis

Since the population of Al Ain did not change over the study periods, the absolute numbers would indirectly reflect the incidence of each mechanism of injury. Accordingly, changes between the two periods were reported in both absolute and relative values. Data are presented as number (%) for categorical data, median (range) for ordinal data and mean (standard deviation) for continuous data. We used Fisher’s exact test to compare categorical data of two independent groups, while the Mann–Whitney *U* test was used for continuous or ordinal data of two independent groups. Statistical Package for the Social Sciences (IBM-SPSS version 26, Chicago, Il) was used for all analyses. A *p* value of less than 0.05 was accepted as significant.

## Results

**Fig. 1:**
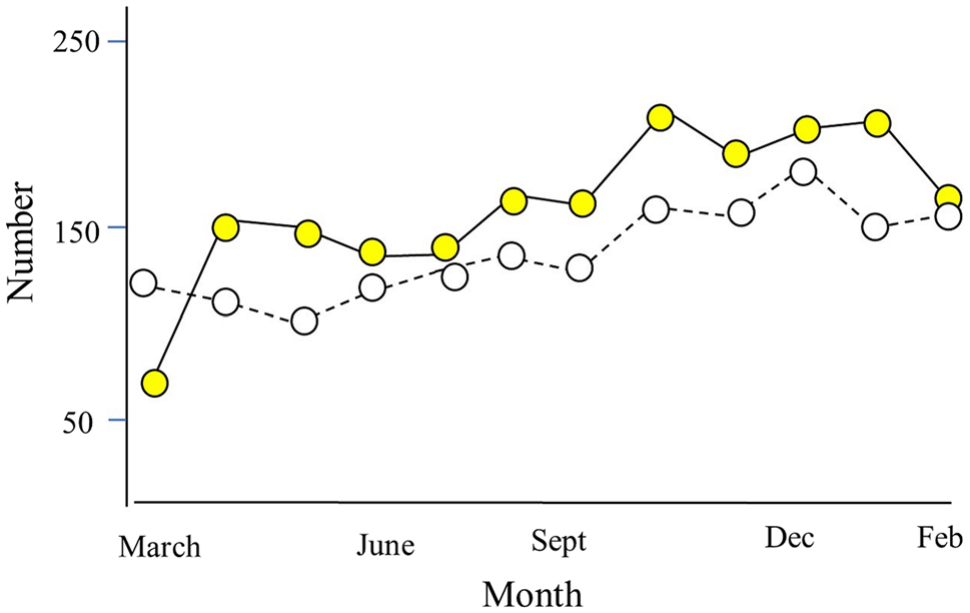
Hospitalised trauma patients during the pre-pandemic period (yellow circles) and pandemic period (white circles), Al-Ain City, United Arab Emirates

Table 1 shows the patients’ demographics and physiological parameters for the two periods. A total of 2002 trauma patients (79.9% males) were admitted in the pre-COVID period, representing an annual trauma incidence of 261.4 trauma patients per 100,000 population. In the second period, a total of 1468 (78.7% male) trauma patients were admitted representing an incidence rate of 191.6/100,000 population and a drop of 26.7% in annual incidence rate. Three patients in the second period tested positive for COVID-19 and were transferred to Al Ain Hospital. All survived to discharge. Figure 1 shows the maximum reduction from March to July 2020 when the government imposed a total lockdown, except for essential services, in the country. There was a rise in rates as the restrictions were lifted during August 2020.

**Table 1:** Demography of hospitalized trauma patients during the pre-pandemic period and the pandemic period, Al-Ain City, United Arab Emirates

Variable	Pre-COVID *n* = 2002	COVID *n* = 1468	*p* value
Age	28 (0–104)	29 (1–96)	0.17
Gender			
Male	1600 (79.9)	1154 (78.7)	0.37
Female	402 (20.1)	313 (21.3)	
Nationality			< 0.001
UAE nationals	881 (44.7)	545 (38)	
Non-UAE	1089 (55.3)	891 (62)	
SBP	132 (0–251)	127 (0–215)	< 0.001
Heart rate	89 (0–199)	91 (0–189)	0.51
Respiratory rate	18 (0–60)	20 (0–48)	< 0.001
GCS*	15 (3–15)	15 (3–15)	0.38
	14.6 (1.8)	14.6 (1.8)	
ISS*	4 (1–75)	4 (1–38)	< 0.001
	6.3 (6.2)	5.6 (5.5)	
ISS > 15	118 (5.9)	87 (5.9)	0.99
Hospital days	2 (1–157)	2 (1–93)	0.79
ICU admit	151 (7.5)	131 (8.9)	0.15
Death	25 (1.2)	27 (1.8)	0.16

Data are presented as median (range) and mean (SD). *p* value Fisher’s exact test or Mann–Whitney test as appropriate

*GCS and *ISS are presented both as median (range) and mean (SD)

Significantly fewer UAE nationals were admitted during the COVID-19 period (38% compared with 44.7%, *p* < 0.001); an absolute reduction of 38%. Patients admitted during the COVID-19 period had significantly lower SBP, higher respiratory rates and lower ISS (*p* < 0.001). There was no significant difference in the hospital length of stay (*p* = 0.79), nor in the overall mortality rates between the two periods (1.2% compared with 1.8%, *p* = 0.16, Fisher’s exact test).

Mechanism of injury significantly differed between the two periods (*p* < 0.0001, Fisher’s exact test). The absolute number of injuries due to machinery and falling objects increased in the pandemic period (39.4% and 54.1%, respectively). In contrast, RTCs and falls were reduced during the pandemic period (33.5% and 31.3%, respectively). Injury due to machinery and falling objects was relatively higher in the pandemic period (7.5% and 3.9% compared with 3.9% and 1.8%, respectively), while RTCs and falls were relatively less during the pandemic period (34.3% and 36% compared with 37.5% and 38.1%, respectively) (Table 2). Table 3 shows the locations of injury. Location significantly differed between the two periods (*p* < 0.0001, Fisher’s exact test). There was an absolute increase of 18.4% in workplace injuries and a reduction of 39.3% in home injuries over the study period. There were relatively more workplace injuries and relatively fewer home injuries during the pandemic period (11.3% and 42.8% compared with 7.1% and 52.4%, respectively).

**Table 2:** Mechanism of injury of hospitalized trauma patients during the pre-pandemic period and pandemic period, Al-Ain City, United Arab Emirates

Mechanism	Pre-COVID *n* = 2002	COVID *n* = 1455	Change (%)
Falls	763 (38.1)	524 (36)	**−** 31.3
RTC	750 (37.5)	499 (34.3)	**−** 33.5
Penetrating	70 (3.5)	70 (4.8)	0
Machinery	78 (3.9)	109 (7.5)	+ 39.7
Burns	46 (2.3)	37 (2.5)	**−** 19.6
Assault	55 (2.7)	52 (3.6)	**−** 5.5
Falling objects	37 (1.8)	57 (3.9)	+ 54.1
Others	203 (10.1)	107 (7.4)	**−** 47.3

Data are presented as number (%), the mark of percentage change indicates an increase (+) or decrease (−). Numbers do not add to the total number of the population because of missing data. Percentages were calculated from available data

**Table 3:** Location of injury of hospitalized trauma patients during the pre-pandemic period and the pandemic period, Al-Ain City, United Arab Emirates

Location*	Pre-COVID *n* = 1913	COVID *n* = 1424	*p* value
Home	1003 (52.4)	609 (42.8)	< 0.001
Highway/street	545 (28.5)	434 (30.5)	
Workplace/farm	136 (7.1)	161 (11.3)	
Public area	61 (3.2)	10 (0.7)	
Others	168 (8.8)	210 (14.7)	

Data are presented as number (%)

*Numbers do not add to the total number of the population because of missing data. Percentages were calculated from available data

## Discussion

This study has shown a 27% reduction in the overall incidence of trauma admissions during the COVID-19 pandemic. Work-related injuries, including falling objects and machinery injuries, were absolutely and relatively more during the pandemic, while RTCs and fall injuries were absolutely and relatively less. There were more workplace injuries and fewer home injuries during the pandemic period. Furthermore, fewer UAE nationals were admitted during the pandemic. However, there was no significant difference in mortality between the two periods.

UAE nationals represent 44.7% of the total trauma admissions in the pre-pandemic period, although they constitute 30% of the population in Al-Ain City [9]. This has been attributed to the difference in risk exposure and behaviour between UAE nationals and non-nationals, mainly in RTCs [10]. During the pandemic period, trauma admissions of UAE nationals reduced by 38.1%. Most of the UAE nationals are governmental office employees, who were asked to work from home, compared with non-nationals who ran basic services and essential work outside homes. Although more people worked from home during the pandemic, there was around a 39% reduction in the number of injured patients that occurred at home. We have previously shown that 39% of trauma admissions in our setting were due to injuries that occurred at home, and that falls on the same level in the elderly and UAE nationals have increased over the last decade [11, 12]. The “stay-at-home” order during the pandemic may have increased the level of supervision for those at risk of falls. The relative increase in work-related injuries from machinery and falling objects are related to increased injuries in non-national workers. Similar to our results, other studies have reported differences in trauma patterns by race and gender during the COVID pandemic [13–15].

Over the study periods, the number of injured patients from RTCs and falls reduced by 33.5% and 31.3%, respectively. This reflects the reduction in person and vehicular movements during the pandemic, especially during March–July 2020 when there was a total lockdown in the Abu Dhabi Emirate with the closure of public facilities and compulsory “stay-at-home” orders by all except essential workers [16]. Many countries worldwide have reported a similar reduction in trauma burden from RTCs and outdoors activities ranging from 20.3 to 84% [17–20].

The two leading causes of trauma deaths in our setting are RTCs (47%) and work-related falls from height [9]. Both of these mechanisms of injury were reduced during the pandemic, while mortality did not change. Although some countries have reported a reduction in trauma mortality rate during the pandemic, others have reported an increase [21]. This may be related to a rise in vehicle speeding and non-compliance with safety rules in the pandemic period. An increase in interpersonal injuries and deliberate self-harm during the pandemic were reported [22, 23]. This could be related to the lockdown of the abusers with their victims and the reluctance of the victims to leave their homes due to the COVID-19 risk [24]. These injuries represent less than 4% at both periods in our study, with no significant change in trend. This is possibly related to supportive family ties and a low incidence of violence in our community.

## Limitations

We have to acknowledge that our study has certain limitations. First, we studied hospitalised trauma patients only in Al-Ain City and not on a national level, which may limit the generalisability of our study to the whole of the UAE. Nevertheless, the laws and regulations during the lockdown were applied nationally, and we think these results may be extrapolated to other cities within the UAE. Second, we may have underestimated the trauma death rates because our registry excludes those who died on the scene. Third, we have to highlight that the sample sizes of each period are relatively small. Despite having lower ISS in the COVID-19 period, mortality was higher (although non-significant). This can be attributed to a delay in the management of trauma patients waiting for the PCR test before admission. This is supported by the fact that those having an ISS of more than 16 were the same between the two periods. Finally, the reduction of trauma incidence over time can be part of the trend of overall improvement in our trauma system, including injury prevention. Mortality of those having severe trauma has dramatically improved over time in our setting [11]. Nevertheless, we were able to demonstrate an immediate rebound increase in trauma following maximum lockdown supports our conclusions.

## Conclusions

The COVID-19 pandemic has modified the trauma risk exposure in our population. It reduced trauma hospital admissions by around 27%. Work-related injuries, including falling objects and machinery injuries were relatively higher during the pandemic. Injury prevention of work-related injuries should be an important component of preparedness for future pandemics.

## Data Availability

There are no additional data available to share with the readers. Data will be shared with the Editor if requested.

## Abbreviations

DOH: Department of health
GCS: Glasgow coma scale
ICU: Intensive care unit
ISS: Injury severity score
RTC: Road traffic collision
RT-PCR: Reverse transcriptase-polymerase chain reaction
SARS-COV-19: Severe acute respiratory syndrome coronal virus-2019
UAE: United Arab Emirates
WHO: World Health Organization
